# Development and Characteristics of Aerated Alkali-Activated Slag Cement Mixed with Zinc Powder

**DOI:** 10.3390/ma14216293

**Published:** 2021-10-22

**Authors:** Taewan Kim, Choonghyun Kang, Kiyoung Seo

**Affiliations:** 1 Department of Civil Engineering, Pusan National University, Busan 46241, Korea; ring2014@naver.com; 2 Department of Ocean Civil Engineering, Gyeongsang National University, Tongyeong 53064, Korea; 3 HK Engineering and Consultants, Busan 46220, Korea; aricari@hanmail.net

**Keywords:** zinc powder, gas agent, alkali-activated slag cement, hydrogen gas, aerated concrete

## Abstract

Experiments on the development and properties of aerated concrete based on alkali-activated slag cement (AASC) and using Zn powder (ZP) as a gas agent were carried out. The experiments were designed for water-binding material (w/b) ratios of 0.35 and 0.45, curing temperatures of 23 ± 2 °C and 40 ± 2 °C, and ZP of 0.25%, 0.50%, 0.75%, and 1.0%. ZP generates hydrogen (H_2_) gas in AASC to form pores. At a w/b of 0.35, the curing temperature had little effect on the pore size by ZP. However, a w/b of 0.45 showed a clear correlation that the pore diameter increased as the curing temperature increased. The low w/b of 0.35 showed a small change in the pore size according to the curing temperature due to the faster setting time than 0.45 and the increased viscosity of the paste. Therefore, at a termination time exceeding at least 60 min and a w/b of 0.45 or more, it was possible to increase the size and expansion force of the pores formed by the ZP through the change of the curing temperature. ZP showed applicability to the manufacture of AASC-based aerated concrete, and the characteristics of foaming according to the curing temperature, w/b ratio, and ZP concentration were confirmed.

## 1. Introduction

Alkali-activated slag cement (AASC) has attracted significant attention as an eco-friendly material compared to ordinary Portland cement (OPC) [1,2,3,4,5,6]. Many studies have shown that AASC has high strength and durability [7,8,9,10]. AASC is widely applicable in various members of construction, and aerated concrete is one of them. Recently, even aerated cement/concrete to which AASC is applied has been researched and developed. Aerated concrete is developed and manufactured to improve the characteristics of cement/concrete, such as weight reduction, water permeability, and thermal insulation. The materials used for foaming are a foaming agent [11,12,13,14], metallic powder (Al, Zn) [15,16,17], or hydrogen peroxide (H_2_O_2_) [18,19,20,21]. Aluminum powder (Al powder) is the most commonly used material for foaming, as mentioned in several previous studies [16,17,22]. Al powder shows low usage and a high foaming effect. The foaming approach applies to a simple method for generating gas to form pores inside a specimen. However, the size, distribution, and quantity of the air foams are affected by various conditions. Several factors, such as binder material, admixture material, curing temperature, mixing ratio, and foaming agent, are considered [23,24,25]. Although aerated concrete can be manufactured quickly and easily, the compressive strength and durability are properties that need improvement. Recently, several studies on aerated concrete using various foaming agents for alkali-activated cement or geopolymer have been conducted [22,24,26,27,28]. For expanded cement/concrete using zinc powder (ZP), studies on OPC-based [23] and fly-ash-based geopolymer [29] or magnesium phosphate [15] have been reported; however, studies on AASC are still insufficient.

Therefore, in this study, an AASC-based experiment was designed to analyze the mechanical properties and bubble formation characteristics of aerated concrete. In this study, considering the high early-age strength characteristic reported as one of the characteristics of AASC, we intend to improve the mechanical characteristics that decrease after bubble formation. Herein, ZP was used as the foaming agent, different from the existing Al powder. It has been reported that the hydrogen gas (H_2_) generated by the reaction of ZP is about half in quantity compared with aluminum powder and causes a relatively slow foaming reaction [29]. However, ZnO is potentially applicable in various fields, and several studies are currently being conducted on the multifunctionality of ZnO in cement/concrete. The zinc oxide applied to cement is a material reported to be effective in antibacterial concrete [30,31], radiation shielding [32], water purification [33], anticorrosion of steel [34], and photocatalysis [35,36].

The Equation (1) shows that ZP powder reacts with water to produce zinc oxide (ZnO) and H_2_. The generated H_2_ expands inside the cement, forming pores.

Zn + H_2_O → ZnO + H_2_ (1)

The ZnO is insoluble in water but soluble in alkaline or acidic environments [37,38]. By examining the various effects of cement using ZnO reported currently, it is expected that the effect of zinc will be improved even more if the application field is expanded and the optimum zinc oxide concentration and mixing conditions are found. Therefore, it is thought that aerated concrete using Zn powder can be broadly applied to multifunctional concrete that basically includes the effect of zinc oxide. For this, it is judged that experiments and studies on the mixing conditions and characteristics of aerated concrete mixed with ZP should be conducted. Furthermore, at the same time, we would like to present the purpose for the development and application of a new metal powder that can replace aluminum powder, which is the existing gas agent used in the production of aerated concrete. AASC has high alkalinity, fast setting, and high strength. Thus, hydration reactants are formed through the dissolution of ZnO in the high alkali environment of AASC. Several studies have been conducted on OPC mixed with ZnO. However, few studies have investigated the application of ZnO to AASC. Moreover, studies on AASC using ZP are rare. A study was recently conducted using 0.3–0.8% ZP in magnesium phosphate cement (MPC) [15]. Additionally, a study exists on porous cement manufacturing using ZP for rapid MPC. Similarly, ZP can be applied to AASC based on the MPC result with rapid setting high strength performance. Therefore, this study has two purposes. The first is the development of aerated cement using AASC, which is attracting attention as an eco-friendly, low-carbon cement. In addition, AASC has faster setting and higher early-age strength than OPC-based cement. This is expected to improve the mechanical performance degradation problem of OPC-based aerated cement to some extent. The second is to examine the influence and characteristics of ZP on aerated AASC. This is because studies on aerated cement using ZP in AASC are very rare. From the results of this experiment, we will examine the development and basic characteristics of aerated AASC using ZP, and plan additional experiments on the size, distribution, and durability of bubbles in subsequent studies. The experiments and analysis on the mechanical properties according to the concentration, formulation, and curing conditions of ZP are first performed. Furthermore, based on the results of the mechanical properties, follow-up studies on antibacterial, photocatalysis, and anticorrosion of steel are planned. For this investigation, the compressive strength, X-ray diffractometer (XRD) spectra, scanning electron microscope (SEM) images, water absorption rate, and ultrasonic pulse velocity (UPV) were measured and analyzed.

## 2. Materials and Methods

### 2.1. Materials

Table 1 shows the results of X-ray fluorescence analysis on the chemical composition of the slag used in this study. For alkali activator, bead type sodium hydroxide (NaOH, purity ≥ 98%) and liquid type sodium silicate (Na_2_SiO_3_, *Ms* = 2.1) were used at a 10% concentration of binder weight (10% NaOH + 10% Na_2_SiO_3_). Before mixing, the activator was added to water, stirred well, and allowed to stand at room temperature in the laboratory for 6 h and then used. The ZP has a gray color, the specific gravity of 7.14, a pH of 6.95–7.37, and an average particle size of 4.0 µm with a purity ≥99.0%.

### 2.2. Experiments

The experimental design considered the effect of three variables: the water-binder ratio (w/b), curing temperature, and ZP content. Here, the binder consists only of 100% slag. Furthermore, the w/b ratio was selected to exclude the effect of superplasticizer. If it is less than w/b = 0.35, mixing and molding of the specimen are difficult, and if w/b = 0.45, material separation occurs due to excessive fluidity. Therefore, the final w/b ratio of 0.35 and 0.45 was selected in the range where the superplasticizer was not used. The authors selected three curing temperatures: 23 ± 2 °C, 40 ± 2 °C, and 60 ± 2 °C through preliminary experiments. However, at a temperature of 60 ± 2 °C, the specimen was not properly formed due to the rapid expansion of ZP. As a result, the curing temperature of the final experimental plan was selected as 23 ± 2 °C and 40 ± 2 °C. Finally, the ZP content was a total of five concentrations, including 0.0%, 0.25%, 0.50%, 0.75%, and 1.00% of the weight of the binder and a mixture without ZP. A total of 20 mixtures were produced. Table 2 summarizes the ratio for the detailed mixture.

Mixing was conducted following the instrument and method of ASTM C305 [39]. The mixed samples were poured into a 50 × 50 × 50 mm^3^ cube metal mold, compacted, and stored in a chamber at 23 ± 2 °C or 40 ± 2 °C and relative humidity (RH) of 90 ± 5% for 24 h. The mold was then removed and stored in the chamber at 23 ± 2 °C and RH of 90 ± 5% until 28 d. The compressive strength was measured after 1, 3, 7, and 28 d, and the average of the measured values of the three samples was used. Microstructural analysis was performed using an XRD with a step size of 0.017° (2θ) from 5° to 60° and a scanning electron microscope/backscattered electron (SEM/BSE) at 15 kV in high vacuum mode. For the physical properties, water absorption rate and UPV were measured.

Table 3 shows the setting time and flow values of the mixture without ZP at 23 ± 2 °C. The setting time was measured following the Gillmore needle test made in ASTM C266 [40]. The flow value of the paste was measured using the flow table apparatus of ASTM C230 [41]. For the mixture with a w/b of 0.35, the initial and final setting times were 20 and 25 min, respectively, and the total setting time is 45 min, setting in less than one hour. However, for the mixture with a w/b of 0.45, the initial and final setting times were 55 and 65 min, respectively, and the total setting time was 120 min, with a more rapid setting. The total setting time of the mixture with a w/b of 0.45 increased about two times compared with that with a w/b of 0.35. This increment is because the slag hydration reaction was delayed due to the dilution of the concentrated activator with the increase in mixed water for the same amount of binder. Moreover, the flow value of the mixture with a w/b of 0.35 was measured as 205 mm, whereas that with a w/b of 0.45 showed excessive fluidity, exceeding the flow table.

Water absorption was measured following the ASTM C1403 [42] method using a 50 × 50 × 50 mm^3^ sample. Dry density was calculated as follows.

$$\rho_{d}=\frac{m_{d}}{V}$$

where *ρd* is the dry density, *m_d_* is the mass of the sample after oven-drying at 105 ± 5 °C for 24 h, and *V* is the volume of the sample.

The UPV was measured on a 40 × 40 × 160 mm^3^ prismatic mold sample. First, the receiver was contacted on the right and the oscillator on the left. Next, the measurement was conducted again with the oscillator on the right and the receiver on the left. The average value of both measurements for a sample was taken as one measurement value. UPV measurement was performed on three samples, and the average value was used as the UPV value.

## 3. Results and Discussion

### 3.1. Compressive Strength

**Figure 1:**
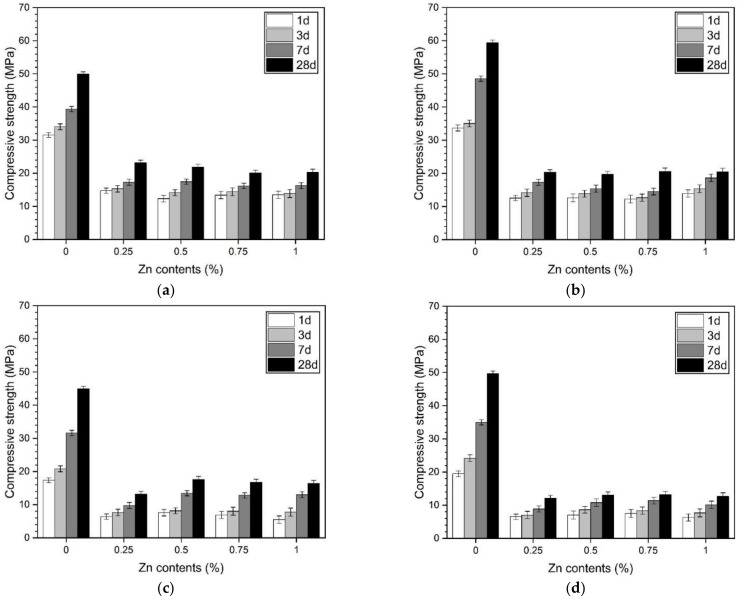
Compressive strength of samples with (**a**) w/b of 0.35, cured at 23 ± 2 °C, (**b**) w/b of 0.35, cured at 40 ± 2 °C, (**c**) w/b of 0.45, cured at 23 ± 2 °C, and (**d**) w/b of 0.45, cured at 40 ± 2 °C.

Figure 1 shows the compressive strength measurement results according to the w/b ratio, curing temperature, and ZP concentration. Regardless of the w/b value and curing temperature, the ZP-containing samples showed low compressive strength values compared with the free-ZP samples. Previous studies have reported that aerated concrete has low compressive strength due to the foamed pores [24].

For the samples with a w/b of 0.35 (Figure 1a,b) and 0.45 (Figure 1c,d), the compressive strength values of the samples with a high w/b were low, with or without ZP. Herein, an increase in the w/b ratio was designed as a mixture in which mixed water increases in the same amount of binder. The concentration of the activator is 10% of the binder weight. Therefore, the mixed-water increment dilutes the concentration of the alkali solution. Consequently, the hydration reaction of the slag is reduced, affecting the decrease in strength. Figure 1a,b shows the results of the compressive strength of the samples with a w/b of 0.35. For the free-ZP samples, the compressive strength at all the measured ages increases as the curing temperature increases, i.e., the strength of the sample cured at 40 °C is more than that cured at 23 °C. However, for the ZP-containing samples, the compressive strength at 40 °C decreased slightly compared with that at 23 °C. The samples with a w/b of 0.45 showed a strong tendency to decrease the compressive strength with an increasing curing temperature (Figure 1c,d). This trend is more significant in the samples with a w/b of 0.45 than those with a w/b of 0.35 as the curing temperature increases. In previous studies of AASC, an increase in the curing temperature promoted the hydration reaction of slag and thus improved the strength. However, by using ZP in this study, no strength improvement was observed.

The decreasing tendency of the compressive strength, even with an increase in the curing temperature, can be considered as follows. ZP reacts with water to generate Zn(OH)_2_ and H_2_ inside the paste. At a curing temperature of 40 °C rather than 23 °C, the samples are set and hardened rapidly. Therefore, the time required for the expansion and movement of the H_2_ is insufficient owing to the rapid setting. However, as shown in Table 3, the sample with a w/b of 0.45 has a longer setting time than the sample with a w/b of 0.35. Therefore, the samples with a w/b of 0.45 have enough time for the bubbles generated by the H2 gas in the paste to move and expand compared to the 0.35 samples. Therefore, the sample with a w/b of 0.45 has reduced strength due to the large diameter pores generated by the ZP. The high w/b ratio and the curing temperature at room temperature increase the setting time of the paste and increase the expansion and movement of the H_2_, acting as a factor to increase the size of the pores.

**Figure 2:**
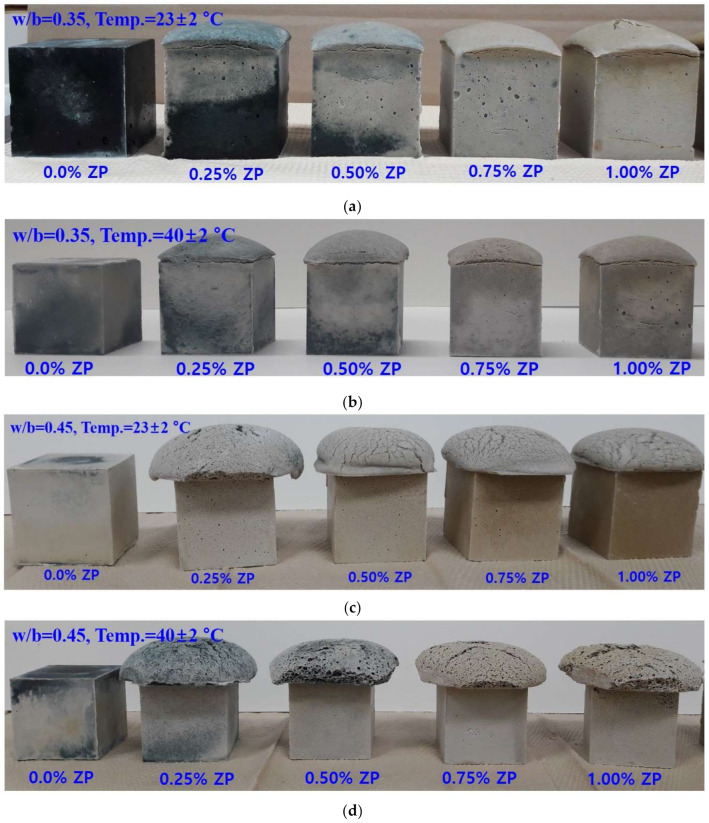
Sample appearances (after 24-h curing) of specimens with (**a**) w/b of 0.35, cured at 23 ± 2 °C, (**b**) w/b of 0.35, cured at 40 ± 2 °C, (**c**) w/b of 0.45, cured at 23 ± 2 °C, and (**d**) w/b of 0.45, cured at 40 ± 2 °C.

Figure 2 shows the appearance of each demolded sample after a 24-h curing. Regardless of the w/b and curing temperature, the top surfaces of the ZP-mixed samples are expanded and swollen. To measure the compressive strength, the expanded part at the top was cut to a size of 50 × 50 × 50 mm^3^ with a low-speed precision cutter. The upper parts of the samples in Figure 2c,d with a w/b of 0.45 are more expanded and swollen than those in Figure 2a,b with a w/b of 0.35. This trend suggests that, as described above, the expansion and movement of the H_2_ caused by the reaction of ZP and water were more active in the samples with a w/b of 0.45 than those with a w/b of 0.35. The expansion height increases as the ZP concentration increases (Figure 2), indicating that, if the ZP amount is doubled (Equation (1)), the expansion height or volume will double as well. However, the ZP amount and the amount of expansion are not proportional. Thus, not all the generated gases are trapped inside the sample as some foams are destroyed or gases escape out of the sample. Such a quantity is difficult to precisely calculate. Similarly, in the study of aerated concrete using H_2_O_2_ as a foaming agent, it was reported that no linear proportional relationship existed between the amount of the foaming agent and the expansion rate [20].

### 3.2. Reaction Products

**Figure 3:**
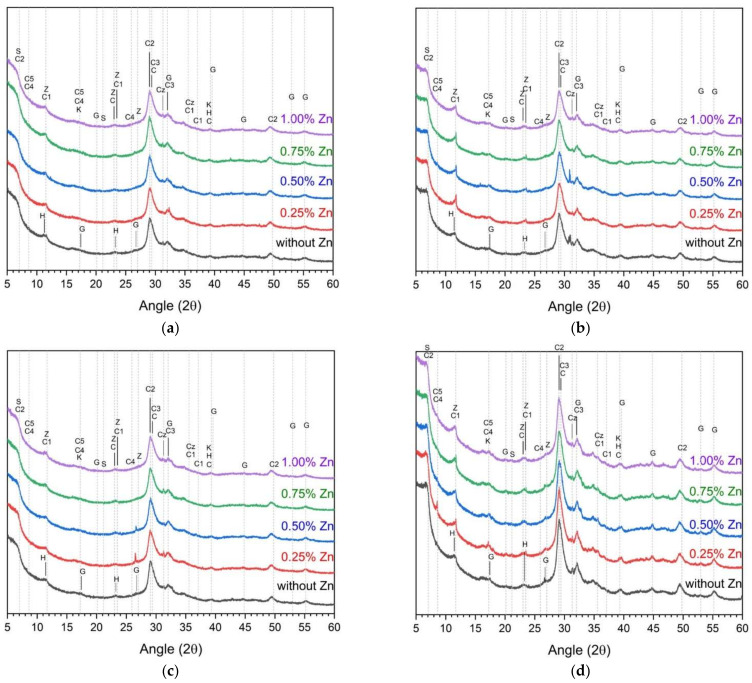
XRD analysis of samples with a w/b of 0.35 (**a**) cured at 23 ± 2 °C after 1 day, (**b**) cured at 23 ± 2 °C after 28 d, (**c**) cured at 40 ± 2 °C after 1 day, and (**d**) cured at 40 ± 2 °C after 28 d.

**Figure 4:**
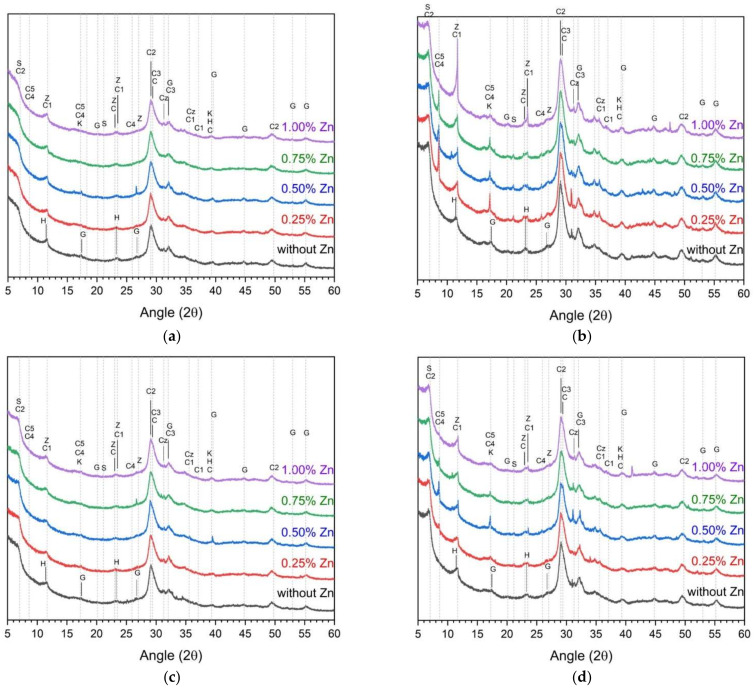
XRD analysis of samples with a w/b of 0.45 (**a**) cured at 23 ± 2 °C after 1 day, (**b**) cured at 23 ± 2 °C after 28 d, (**c**) cured at 40 ± 2 °C after 1 day, and (**d**) cured at 40 ± 2 °C after 28 d.

Figure 3 and Figure 4 show the XRD analysis results for determining the hydration reactants according to the w/b ratio, curing temperature, and ZP concentration. In the ZP-free samples, hydrotalcite, stratlingite, hydroganet, monocarboaluminate, C–S–H(I), C–S–H gel, calcite, and katoite were observed regardless of the w/b [9,28,43,44,45]. Table 4 summarizes the classification and types of the hydration reactants shown in Figure 3 and Figure 4.

Moreover, after 28 d as compared to one day, the slag hydration reaction increased and the peak heights of the stratlingite, hydrogarnet, C–S–H(I), and C–S–H increased. In the ZP-containing samples, calcium aluminum oxide sulfate hydrate, calcium iron sulfate hydrate, calcium ZnO, and zinc hydroxide were additionally observed. Precisely, calcium zinc oxide and zinc hydroxide are hydration reactants found in previous studies involving ZnO [36,46,47].

It has been reported that the crystalline phase of calcium zincate (CaZn_2_(OH)_6_·2H_2_O, wurtzite type) reduces the pozzolanic reaction by consuming the Ca(OH)_2_ [48,49]. However, since there is no Ca(OH)_2_ in AASC, it can be considered that the calcium zincate is formed by the presence of calcium eluted from the slag and OH-ions supplied from the activator. Other studies have reported that the ZnO interferes with the C–S–H-gel formation. ZP reacts with water to generate ZnO and H_2_ gas (Equation (1)). ZnO forms Zn-based hydrates through the following reactions (Equations (2)–(4)). In the reactions up to Equations (2)–(4), the reaction proceeds by OH^−^ supplied from the alkali activator and calcium ions eluted from the slag [34,46,50,51]. Precisely, the environment in which OH^−^ ions are sufficiently supplied by the activator induces the reactions up to Equations (2)–(4) quickly.

ZnO + H_2_O → Zn^2+^ + 2OH^−^ → Zn(OH)_2_ (2)

ZnO + H_2_O + 2OH^−^ → Zn(OH)_4_^2−^ (3)

2Zn(OH)_4_^2−^ + Ca^2+^ + H_2_O → Ca(Zn(OH)_3_)_2_·H_2_O + 2OH^−^ (4)

The addition of ZP slightly affected the hydration reaction product of AASC. No remarkable peak change of the hydration reaction product nor the formation of a new hydration reaction product was observed due to the change in the ZP concentration.

### 3.3. Microstructures

**Figure 5:**
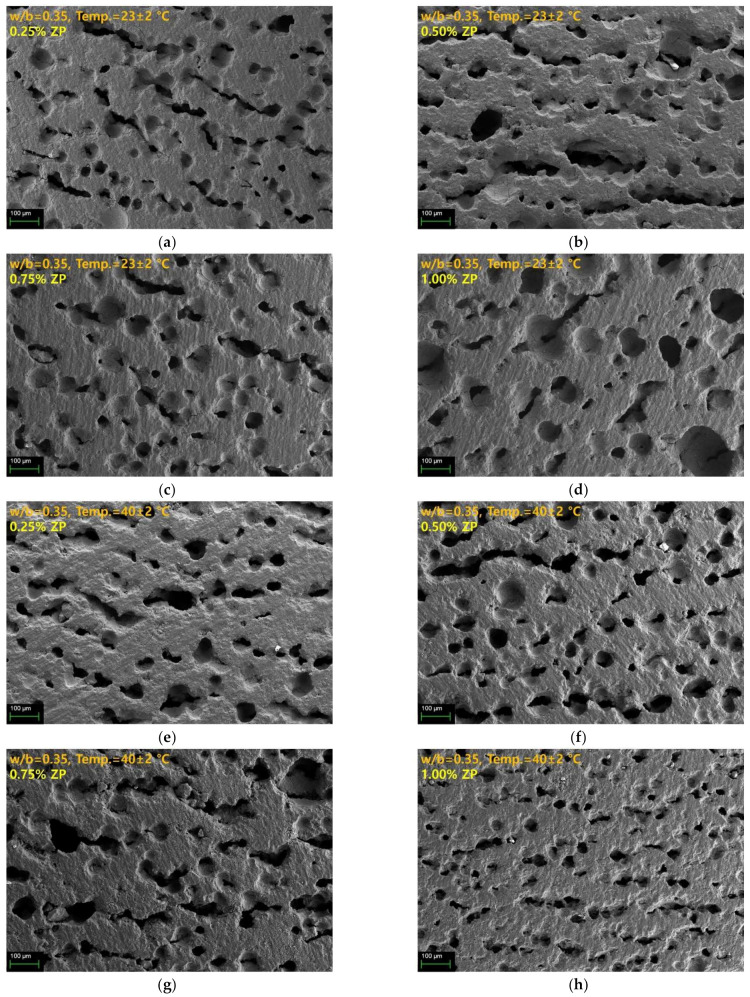
SEM images of samples with a w/b of 0.35 (**a**) cured at 23 ± 2 °C, 0.25% ZP, (**b**) cured at 23 ± 2 °C, 0.50% ZP, (**c**) cured at 23 ± 2 °C, 0.75% ZP, (**d**) cured at 23 ± 2 °C, 1.00% ZP, (**e**) cured at 40 ± 2 °C, 0.25% ZP, (**f**) cured at 40 ± 2 °C, 0.50% ZP, (**g**) cured at 40 ± 2 °C, 0.75% ZP, and (**h**) cured at 40 ± 2 °C, 1.00% ZP.

**Figure 6:**
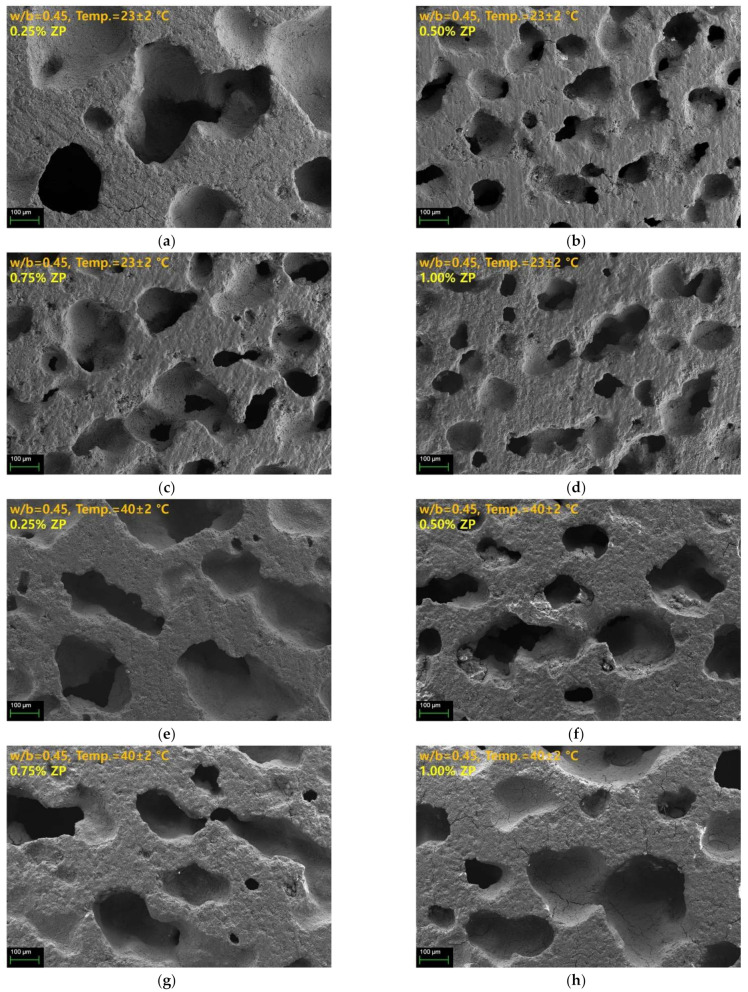
SEM images of samples with a w/b of 0.45 (**a**) cured at 23 ± 2 °C, 0.25% ZP, (**b**) cured at 23 ± 2 °C, 0.50% ZP, (**c**) cured at 23 ± 2 °C, 0.75% ZP, (**d**) cured at 23 ± 2 °C, 1.00% ZP, (**e**) cured at 40 ± 2 °C, 0.25% ZP, (**f**) cured at 40 ± 2 °C, 0.50% ZP, (**g**) cured at 40 ± 2 °C, 0.75% ZP, and (**h**) cured at 40 ± 2 °C, 1.00% ZP.

Figure 5 and Figure 6 show SEM images according to the w/b and curing temperature. The shapes of the foams observed in Figure 5 and Figure 6 are not completely spherical but elliptical, formed by aeration [52,53]. Figure 5a–d shows the cross-sectional SEM images of the samples cured at 23 °C, with a ZP concentration of 0.25–1.00%. Several voids were observed in the cross-sectional view, and some were connected. Even with increased ZP concentration, the diameters of the pores did not change significantly, showing similar sizes. Figure 5e–f shows the cross-sectional SEM images of the samples cured at 40 °C, with similar pore sizes and distributions regardless of the ZP concentration. The number of pores of such samples slightly increased compared with those cured at 23 °C.

Figure 6 shows the cross-sectional SEM images of the samples with a w/b of 0.45 according to the curing temperature. Figure 6a–h show the cross-sectional SEM images of the samples cured at 25 °C and 40 °C, respectively. The diameters of the pores observed in the latter increased more than those observed in the former. Although the diameters of the pores increased, the number of pores decreased. Besides, the diameters of the samples with a w/b of 0.45 are 2–3 times larger than those with a w/b of 0.35, which agrees with the results of previous studies where the foam content increased as the w/b increased [26].

The pore diameter and distribution characteristics observed in the cross-sectional SEM images of the samples seem to be significantly affected by the w/b and curing temperature rather than the ZP concentration. A high w/b and low curing temperatures delay the slag hydration reaction and increase the setting time. This phenomenon gives sufficient time for the H_2_ generated by the reaction of ZP and water to expand and move. Consequently, the pores present in the samples with a w/b of 0.45 are larger than the pores present in the samples with a w/b of 0.35. Besides, with a w/b ratio of 0.45, the pore diameter was larger for the samples cured at 40 °C than at 25 °C despite an increase in the curing temperature. Thus, the increase in the temperature affects the expansion of the H_2_. However, for the samples with a w/b of 0.35, the pore diameter change according to the curing temperature was different from those with a w/b of 0.45. The samples with a w/b of 0.35 showed small-sized pores due to the fast-setting time, even at 25 °C. Increasing the curing temperature to 40 °C further inhibited the expansion and movement of the voids due to a faster setting. Consequently, with a w/b of 0.35, the difference in the pore diameter based on curing temperatures of 25 °C and 40 °C was insignificant. Despite increasing the curing temperature, the setting speed was faster than the expansion speed of the H_2_, indicating that the pore diameter did not increase. To change the pore diameter by ZP, an appropriate setting time for smooth H_2_ expansion and movement is necessary.

It has been reported in previous studies that an increase in the alkali activator concentration reduces the curing time, consequently reducing the foam pores’ size [29]. Therefore, the rapid setting and hardening of AASC suppresses gas expansion. As a result, the size of the pores formed inside the specimen becomes smaller. The amount of the air foams increases as the amount of ZP increases, although the difference is insignificant. The difference in the amount and size of the air foam according to the amount of ZP is insignificant (Figure 5), indicating that, as the ZP increases, the number of foams increases. As the foams merge and the liquid film thickens, the size of the small foams increases, and, simultaneously, the breakdown of the foams increases [26,28]. However, the consolidation and destruction of these foams were difficult to observe in insufficiently grown foams due to the rapid setting time of the AASC. Therefore, when the w/b ratio of 0.45 is compared with that of 0.35, the consolidation of the air foams and the size increase are observed due to higher fluidity and longer setting time. This phenomenon becomes clear by comparing Figure 5 and Figure 6. The increase in the foam content affects the decrease in compressive strength [26].

### 3.4. Water Absorption and Dry Density

**Figure 7:**
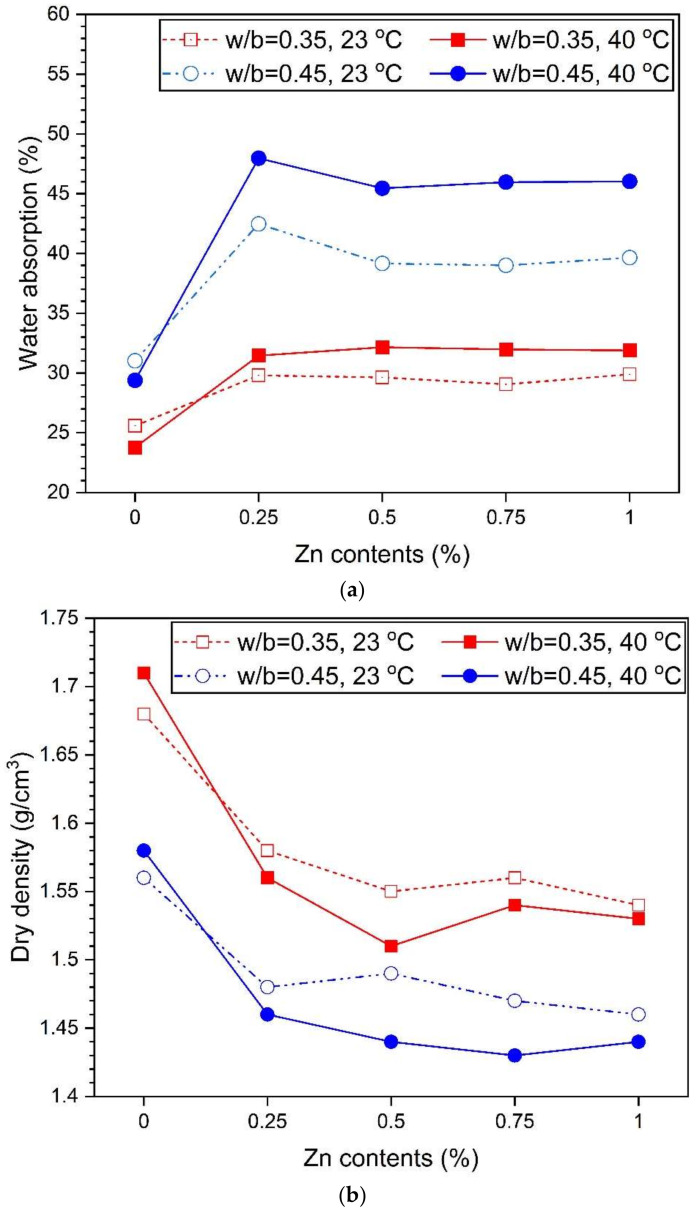

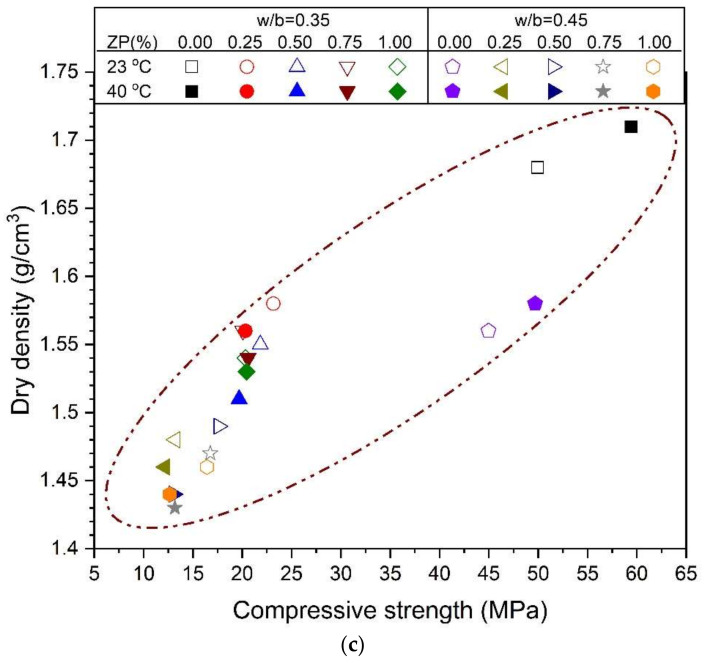
Water absorption and UPV variation with Zn content—(**a**) water absorption, (**b**) dry density, and (**c**) dry density vs. compressive strength.

Figure 7 shows the results of measuring the water absorption and ultrasonic pulse rates. The water absorption rate (Figure 7a) increased as the ZP concentration increased regardless of the w/b ratio and curing temperature. At a w/b of 0.35, the sample without ZP decreased from 25.58% to 23.78% when the curing temperature increased from 23 °C to 40 °C. Moreover, even with a w/b of 0.45, the water absorption rate of the sample without ZP decreased from 31.02% to 29.38% when the curing temperature increased from 23 °C to 40 °C.

With a w/b of 0.35, the difference between the absorption rates between the curing temperatures of 23 °C and 40 °C was <3%. The pore diameters and distribution were similar regardless of the curing temperature (Figure 5). Consequently, the difference in the absorption rate is also small. However, with a w/b of 0.45, the absorption rate was 5.5–7.0% at curing temperatures of 23 °C and 40 °C, showing a greater difference than with a w/b of 0.35. This trend shows the difference in the pore diameter based on the curing temperature (Figure 6). Thus, the sample cured at 40 °C has a larger pore diameter than that cured at 23 °C, indicating an increase in the water absorption space.

Figure 7b shows that the dry density is higher in the samples with a w/b of 0.35 than those with a w/b of 0.45. As shown in Figure 5 and Figure 6, since the inner pore size of the samples with a w/b of 0.45 is more than that with a w/b of 0.35, the density is relatively smaller. According to previous studies, as the amount of the foaming agent increases, the dry density decreases. In Figure 7b, the samples with a w/b of 0.45 show similar trends to the results of previous studies. However, the sample with a w/b of 0.35 has the lowest density at 0.5% ZP, which increases slightly again at 0.75% and 1.0% ZP. This increment may be attributed to the merging and collapsing effects of the void structures [22,54]. In previous studies, it was reported that the compressive strength increases as the dry density increases [22,26]. Figure 7c shows the relationship between the dry density and compressive strength. The dry density and compressive strength were linearly proportional. That is, the higher the dry density, the higher the compressive strength. The results of Figure 7c are consistent with those of previous studies on aerated concrete that reported a linearly proportional relationship between the density and compressive strength [15,24].

### 3.5. Ultrasonic Pulse Velocity (UPV)

**Figure 8:**
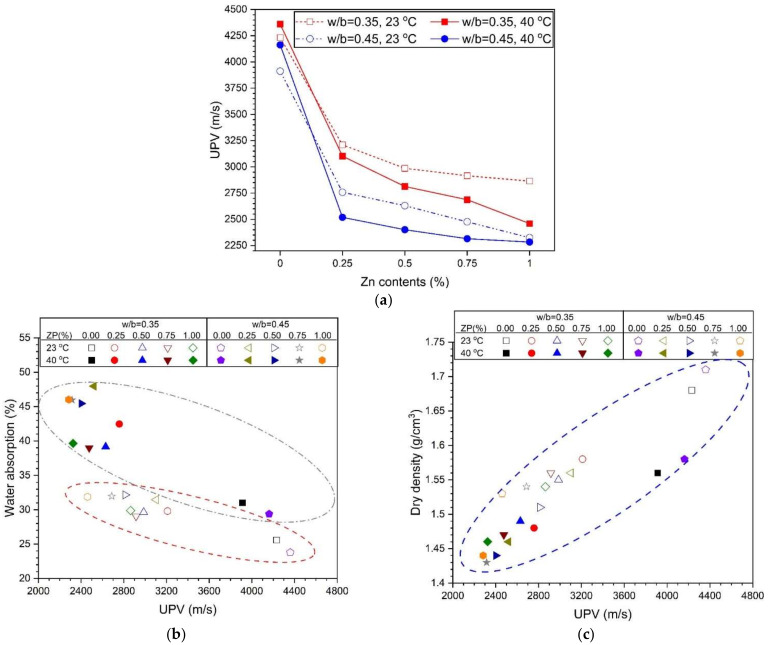
(**a**) Variations of UPV with Zn content, (**b**) water absorption with UPV, and (**c**) dry density with UPV.

Figure 8 shows the measurement results of the UPV. For the ZP-free samples, the UPV also increases as the curing temperature increases from 23 °C to 40 °C, regardless of the w/b. However, as the ZP content increases, the UPV decreases, which may be attributed to the decrease in the voids in the matrix. Just as the difference in the absorption rate based on the curing temperature was insignificant and large with a w/b of 0.35 and 0.45, respectively, the difference in the UPV between the curing temperatures was larger with a w/b of 0.45 than that with a w/b of 0.35.

The similarity between the water absorption rate and the UPV based on the w/b ratio and the curing temperature is examined in Figure 8b. A linear inverse relationship was established between the absorption rate and UPV. With a w/b of 0.35 and 0.45, the absorption rate and UPV are located in the upper left and lower right corners, respectively. An increase in the w/b indicates a high absorption rate and a low UPV. Figure 8c shows the correlation between the dry density and UPV. The dry density-UPV shows the relationship between the water absorption and UPV. The UPV increases as dry density increases, indicating a small number of voids. Therefore, increasing the ZP concentration decreases the dry density and UPV regardless of the w/b because voids are formed by H_2_ generation due to the ZP mixing. Furthermore, as mentioned in the SEM of Figure 5 and Figure 6, the samples with a w/b of 0.45 had larger pores than those with a w/b of 0.35. The density and UPV of the samples with a w/b of 0.45 were smaller than those with a w/b of 0.35.

## 4. Conclusions

The results of examining the strength and microstructural properties of AASC mixed with zinc powder (ZP) at a concentration of 0.25–1.0% are summarized as follows.

- ZP reduces the compressive strength regardless of the w/b and curing temperature. ZP reacts with water to generate H2 to form several pores inside the sample, causing a sharp decrease in the strength. Compared to the sample without ZP, a strength decrease of about 50% is observed. The larger the w/b ratio (0.45 > 0.35), the curing temperature (40 ± 2 °C > 23 ± 2 °C), and the concentration of ZP (1.0 > 0.75 > 0.5 > 0.25 > 0.0), the more the diameter of the bubbles formed inside the sample increases, which is a direct factor in the strength.
- The new hydration reactants by ZP are calcium zincate (Wurtzite type) and zinc hydroxide. No new hydration reactants were observed according to the concentration of ZP, w/b ratio, and curing temperature. In all the samples, the increase and sharpening of the C-S-H peaks over time were most pronounced. The decrease in the mechanical performance due to the formation of bubbles is a greater influence factor than the change in the hydration reactant due to the mixing of ZP.
- Increasing the ZP content increases the water absorption and decreases the dry density and UPV. Moreover, the difference in characteristics according to the w/b ratio and the curing temperature was clearly shown. Compared with a w/b = 0.35, at 0.45, the difference in the water absorption, dry density, and UPV according to the curing temperature was relatively large. The increase in the w/b ratio shows the effect of the diameter and distribution of the pores formed by ZP on the absorption rate, density, and UPV of the sample.
- The bubble formation characteristics according to the curing temperature show different aspects according to the w/b. The w/b = 0.35 had little effect on the pore size and distribution of the samples cured at 23 ± 2 °C and 40 ± 2 °C. However, in the case of W / B in 0.45, the difference in the pore diameter according to the curing temperature was significant. That is, it was observed that the pore diameter becomes larger at 40 ± 2 °C than 23 ± 2 °C. This tendency is that, the smaller the w/b ratio, the shorter the setting time, which is considered to be a factor limiting the expansion and movement of the pores by the H_2_ gas.
- Zinc powder suggested the possibility of being used as a gas for the production of aerated concrete instead of aluminum powder. To control the diameters and distribution of the pores by ZP, it is necessary to increase the curing time of the AASC paste. The setting time or curing time is considered to be one of the important factors in controlling the movement and expansion of the H_2_ generated by ZP. In addition, the construction site construction at room temperature (23 ± 2 °C) and at a high temperature (40 ± 2 °C) are expected to be fully utilized for manufacturing concrete products in factories.

## Figures and Tables

**Table 1:** Chemical components and physical properties used in slag.

	Chemical Components (%)							Density (g/cm^3^)	Fineness (cm^2^/kg)	LOI (%)
	SiO_2_	Al_2_O	Fe_2_O_3_	MgO	CaO	K_2_O	SO_3_			
Slag	34.57	10.88	0.61	4.19	44.56	0.37	3.94	2.89	4200	0.96

**Table 2:** Mix properties.

w/b	Curing Temperature (°C)	ZP Contents (%)
0.35	23 ± 2 °C	0.00
		0.25
		0.50
		0.75
		1.00
0.45		0.00
		0.25
		0.50
		0.75
		1.00
0.35	40 ± 2 °C	0.00
		0.25
		0.50
		0.75
		1.00
0.45		0.00
		0.25
		0.50
		0.75
		1.00

**Table 3:** Setting time and flow value of the mixture without ZP (23 ± 2 °C).

w/b	Setting Time (min)		Flow Value (mm)
	Initial	Final	
0.35	20	25	205
0.45	55	65	overflow

**Table 4:** Hydration reaction summary (in Figure 3 and Figure 4).

Label	Hydration Reactant
C	calcite
C1	monocarboaluminate
C2	C-S-H(I)
C3	C-S-H gel
C4	calcium aluminum oxide sulfate hydrate
C5	calcium iron sulfate hydrate
H	Hydrotalcite
K	Katoite
S	Stratlingite
G	Hydrogarnet
Cz	Calcium zinc oxide (Wurzite type)
Z	Zinc hydroxide

## Data Availability

Data sharing is not applicable to this article.
